# Moxibustion delays ovarian aging by regulating mitochondrial biogenesis and improving oocyte quality

**DOI:** 10.1186/s13020-026-01375-3

**Published:** 2026-04-10

**Authors:** Yaoli Yin, Yan Xu, Zemin Li, Meilin Chen, Ziwei Song, Hongxiao Li, Ge Lu, Meihong Shen

**Affiliations:** 1 College of Acupuncture Moxibustion and Tuina, Nanjing University of Chinese Medicine, Nanjing, China; 2 Key Laboratory of Acupuncture and Medicine Research of Ministry of Education, Nanjing University of Chinese Medicine, Nanjing, China; 3 Institute of Acupuncture and Moxibustion, China Academy of Chinese Medical Sciences, Beijing, China

**Keywords:** Mitochondrial biogenesis, Ovarian aging, Oocyte, Moxibustion

## Abstract

**Background:**

The growing trend of delayed childbearing in contemporary society has made fertility preservation a significant issue, prompting the search for diverse therapeutic options. Conversely, moxibustion is gaining increasing attention as a potential non-pharmacological therapy for supporting reproductive health.

**Methods:**

Mice aged 2 to 14 months were assessed for ovarian function detection to determine the age of reproductive senescence and to identify the optimal time for moxibustion intervention to delay senescence. Oocyte quality and mitochondrial function assessments were conducted to investigate the role of mitochondrial biogenesis in ovarian aging and the underlying mechanisms following moxibustion intervention.

**Results:**

Mice aged 10 months demonstrated ovarian dysfunction associated with aging. Moxibustion significantly elevated hormone levels, increased the number of growing follicles, enhanced embryo implantation and viable birth rates, and reduced embryonic mortality in aging mice. The most pronounced effects were observed in 10-month-old mice. These beneficial outcomes might be linked to improved oocyte quality. Crucially, moxibustion positively increased mitochondrial quantity, enhanced mitochondrial quality, and influenced mitochondrial biogenesis, an effect comparable to that of the peroxisome proliferator‐activated receptor γ coactivator 1α (PGC-1α) agonist valproic acid. Furthermore, the beneficial effects of moxibustion were partially attenuated by a PGC-1α inhibitor.

**Conclusions:**

Moxibustion, as a non-pharmacological intervention, may mitigate ovarian aging and serve as an effective therapeutic strategy for extending human fertility.

**Graphical Abstract:**

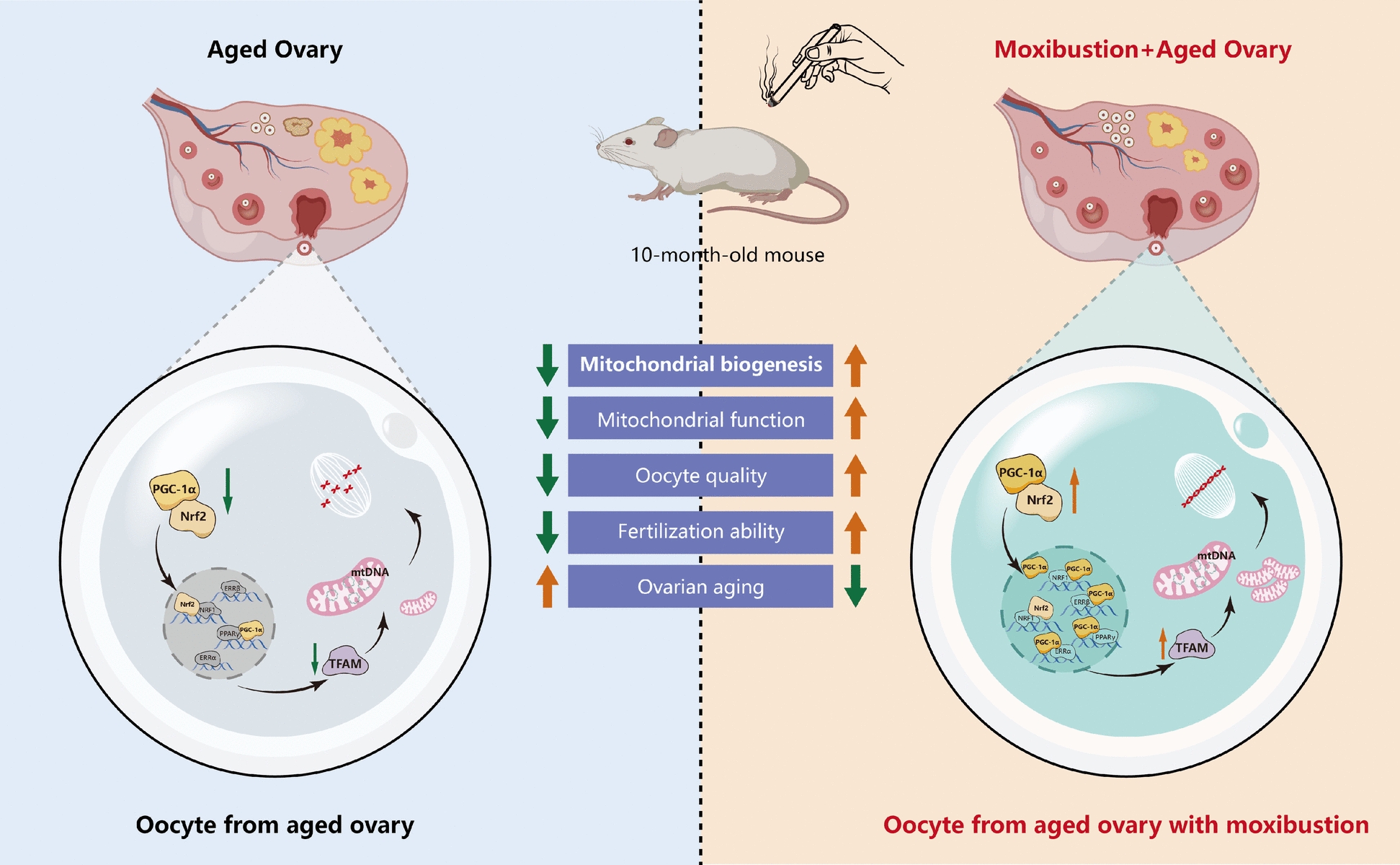

**Supplementary Information:**

The online version contains supplementary material available at 10.1186/s13020-026-01375-3.

## Introduction

In contemporary society, the trend towards delayed childbearing has become widespread. Age-related ovarian aging contributes to an increased incidence of infertility, miscarriage, and adverse neonatal outcomes [1, 2]. The maintenance of fertility in women with advanced maternal age has become an important social issue. Although assisted reproductive technology (ART) partially addresses the demand for delayed fertility, it does not resolve the underlying problems of age-related ovarian senescence and declining oocyte quality, which are the primary causes of female reproductive aging. Early intervention to improve oocyte quality, slow or stop the process of ovarian aging [3], and preserve women's reproductive endocrine function and fertility is particularly important for maintaining physical and reproductive health.

A growing body of evidence has highlighted that mitochondrial dysfunction is a major cause of the decline in oocyte quality [4–6]. Oocyte growth and maturation are highly dependent on mitochondrial function. With age, the oocyte mitochondria undergo abnormal morphological and functional changes. Compared with those in young, the number of mitochondria in the oocytes of aged mice is reduced and unevenly distributed, spindle assembly defects and chromosome misalignments occur [7], mitochondrial membrane potential (MMP) significantly reduces [8], and abnormal mitochondrial morphology with swelling or vacuolization is observable under electron microscopy [9]. Modulation of oocyte mitochondrial distribution and enhancement of mitochondrial activity significantly ameliorate age-related declines in oocyte quality and fertility [9, 10].

Mitochondrial biogenesis plays a central role in regulating mitochondrial function. In women with advanced maternal age, the oocyte mtDNA content is significantly lower than that in younger women [11, 12]. Moreover, impaired oocyte quality due to insufficient mitochondrial content may prematurely induce compensatory activation of mitochondrial biogenesis, which in turn leads to embryonic developmental failure [13]. The activation of mitochondrial biogenesis during oocyte maturation to compensate for mitochondrial depletion and energy deficiency can rescue early embryonic development [13, 14]. Activation of mitochondrial biogenesis to increase the number of mitochondria may be an important therapeutic strategy for treating mitochondrial dysfunction.

Recent evidence suggests that moxibustion, a traditional Chinese medicine derived from clinical practice, can delay organ aging and address various health issues, particularly declining ovarian function. For example, it improves ovarian function by inhibiting apoptotic events and upregulating antioxidant defense mechanisms in naturally aged ovaries [15]. Our preliminary studies revealed that moxibustion ameliorated chemotherapy-induced ovarian aging by mitigating mitochondrial dysfunction [16]. However, the precise mechanism by which moxibustion improves mitochondrial function and delays ovarian aging remains unclear. Moreover, the optimal timing of moxibustion intervention, which is a critical factor for clinical efficacy, remains to be determined. To address these questions, this study compared ovarian function in mice aged 2, 6, 10, and 14 months to identify the appropriate intervention window and investigate the potential role of mitochondrial biogenesis in moxibustion-mediated ovarian improvement.

## Materials and methods

### Animals

Two-month-old female and male mice (20 ± 2 g) were purchased from Hangzhou Medical College (Hangzhou, China), License No.: SCXK (ZHE) 2010–0002. They were routinely kept in a well-ventilated environment with a 12h:12h light/dark cycle, a room temperature of 24–26℃, and a humidity of 60–70%. The mice had free access to drinking water and were fed with full-value pellet diets for acclimatization for 3 d. The disposition of the animals during the experiment was in accordance with the Guiding Principles of Animal Research in China and followed the relevant provisions of the Guiding Opinions on the Kind Treatment of Laboratory Animals, which was approved by the Ethics Committee of the Laboratory Animal Center of Nanjing University of Chinese Medicine, with ethical application numbers 202210A040, 202210A041, and 202304A053. Details of the experimental groups are shown in supplementary material Additional file 1.

### Moxibustion protocol

Based on a previous study [17], two sets of acupoints were used for moxibustion intervention in this study: the bilateral BL23 and CV4 and CV12. Moxibustion was applied to one group of acupoints each day for 10 min; burning moxa sticks (diameter: 4.3 mm; length: 85 mm) were positioned 10 mm above the acupoints. Moxibustion was administered once daily for 21 consecutive days.

### Estrous cycle examination

All female mice underwent estrous cycle monitoring at 8:00 am using vaginal smear analysis. The predominant cell type was determined by microscopic identification. The normal estrous cycle recurs every 4–5 d and is characterized by four stages: proestrus (P), estrus (E), metestrus (M), and diestrus (D).

### Tissue collection and histological analysis

The female mice were euthanized by cervical dislocation. The ovaries were immediately collected and fixed in 4% paraformaldehyde for 48 h, followed by trimming and dehydrating using a graded ethanol and xylene series, and embedded in paraffin. The paraffin blocks were then sectioned at a thickness of 6 μm, and one out of ten sections was stained with HE to evaluate the number of follicles and ovarian morphology.

### Ovarian tissue aging detection

Immunohistochemical (IHC) staining was performed to assess ovarian tissue aging. Paraffin-embedded ovarian tissue sections were subjected to deparaffinization, endogenous peroxidase inactivation, antigen retrieval, and blocking with 5% BSA. The sections were then incubated overnight at 4 ℃ with the following primary antibodies: rabbit anti-P16 (T55316S, Abmart, 1:400) and rabbit anti-P21 (T55543S, Abmart, 1:400). After rewarming, a secondary antibody was applied, followed by incubation with SABC reagent. Chromogenic development was terminated when a light yellow or brown-yellow stains was visible. Finally, sections were counterstained, dehydrated, and mounted. Five randomly selected fields per section were imaged under a light microscope at × 200 magnification; the average optical density was quantified via ImageJ software (Version 1.51j8).

### Hormone levels measurement

On the day of diestrus, female mice were euthanized for serum collection. The serum levels of follicle stimulating hormone (FSH), E_2_, and anti-Müllerian hormone (AMH) were assessed by ELISA according to the manufacturer’s instructions (E-EL-M0511, E-OSEL-M0008, E-EL-M3015, Elabscience). For intra-assay precision, the coefficient of variation was maintained at less than 10%.

### Fertility test

Female mice at the estrous stage were chosen to mate with male mice with proven fertility. Live and abnormal fetuses were identified at E18.5.

### MII oocyte collection

Mice were first injected with 10 IU pregnant mare serum gonadotropin (PMSG, Ningbo Second Hormone Factory), followed by a single injection of 5 IU human chorionic gonadotropin (hCG; Ningbo Second Hormone Factory) 48 h later. Cumulus–oocyte complexes were collected from the fallopian tubes after 14–16 h of hCG injection and placed in M2 medium containing 0.1% hyaluronidase (H3506, Sigma), with several washes to remove cumulus cells.

### Oocyte spindle and chromosome morphology evaluation

The oocytes were fixed with 4% paraformaldehyde for 20 min at room temperature, and their membranes were permeabilized with 0.1% Triton X-100 for 15 min. After blocking for 1 h, the oocytes were incubated with the mouse anti-Alpha Tubulin (66031–1-Ig, Proteintech, 1:4000) at 37℃ for 1 h, and multiple immunofluorescence staining was performed using a tyramide signal amplification-based kit (AFIH C023, AiFang biological technology), according to the manufacturer’s instructions. The oocytes were stained with Hoechst 33342 (C1027, Beyotime Biotechnology) for 10 min at room temperature. Images were recorded using a confocal microscope (TCS SP8, Leica). The excitation/emission wavelengths used for TYR-520 and Hoechst 33,342 were 490/520 nm and 350/461 nm, respectively, and were analyzed using ImageJ software.

### Reverse transcription quantitative PCR

The total RNA was extracted from oocytes using a Single Cell Sequence Specific Amplification Kit (Vazyme, Nanjing, China). To eliminate potential genomic DNA contamination, all RNA samples were treated with DNase I. RT-qPCR was carried out using Hieff UNICON® Universal Blue qPCR SYBR Green Master Mix (Yeasen, Shanghai, China) using an Mx3000P qPCR (Agilent, Beijing, China). The qPCR primers were designed by Sangon Biotech (Shanghai, China), and the sequences are presented in Additional file 2. The specificity of each amplification was verified by melting curve analysis. The relative mRNA expression levels were calculated using the comparative 2^−△△CT^ method [18].

### Mitochondrial function assessment

Ten oocytes in each group were collected to determine ATP concentration using an Enhanced ATP Assay Kit (S0027, Beyotime Biotechnology), following the manufacturer’s instructions.

Mitochondrial distribution and reactive oxygen species (ROS) in the mitochondria were evaluated using MitoTracker Green (C1048, Beyotime Biotechnology) and MitoSOX Red Mitochondrial Superoxide Indicator (40778ES50, Yeasen) staining. The oocytes were labeled with 200nM Mito-Tracker Green for 30 min at 37 ℃. Following careful washing in M2 medium, the oocytes were then incubated in 5μM MitoSOX Red Mitochondrial Superoxide Indicator for 30 min and 10 × Hoechst 33342 for 10 min at 37 ℃, respectively. The oocytes were then observed under a confocal microscope (TCS SP8; Leica, Wetzlar, Germany) after washing with M2 medium. The excitation/emission wavelengths used for MitoTracker Green and MitoSOX Red were 490/516 nm and 510/580 nm, respectively. MMP (Ψm) was measured through an enhanced MMP assay kit with JC-1 (C2003S, Beyotime Biotechnology); the excitation/emission spectra used for J-aggregates and monomers were 525/590 nm and 490/530 nm, respectively. The intensity of each fluorescence signal was analyzed using the ImageJ software.

### Oocyte immunofluorescence

The oocytes were fixed with 4% paraformaldehyde for 20 min at room temperature, and their membranes were permeabilized with 0.1% Triton X-100 for 15 min. After blocking for 1 h, the oocytes were incubated with the following primary antibodies at 37℃ for 1 h: mouse anti-PGC-1α (66369–1-Ig, Proteintech, 1:200) and rabbit anti-Nrf2 (16396–1-AP, Proteintech, 1:200). The immunofluorescent signals were labeled with the following fluorescent secondary antibodies at room temperature for 1 h: CoraLite594–conjugated Goat Anti-Mouse IgG(H + L) (SA00013-3, Proteintech, 1:250) and CoraLite488-conjugated Goat Anti-Rabbit IgG(H + L) (SA00013-2, Proteintech, 1:250), and they were counterstained with Hoechst 33,342 for 10 min. Images were recorded using a confocal microscope (TCS SP8, Leica), and the excitation/emission wavelengths used for CoraLite594, CoraLite488, and Hoechst 33,342 were 593/614 nm, 488/515 nm, and 350/461 nm, respectively, and were analyzed using ImageJ software.

### Mitochondrial morphology assessment

The oocytes were fixed with 2.5% glutaraldehyde for 2 h and stained with eosin (Phygene, PH0500) for 5–10 s and then embedded in 1.5% agarose (Biosharp, BS081). In a 2 × 2 × 2 mm agarose gel cube, the oocytes were fixed in 2.5% glutaraldehyde at 4 ℃ overnight and then wrapped in epoxypropane resin following the standard procedure for transmission electron microscopy. Oocyte mitochondria were observed under a transmission electron microscope (JEM-1400Flash, JEOL).

### Statistical analysis

All the data were analyzed via IBM SPSS Statistics (version 26.0, SPSS Inc.). Regarding the data that met both normal distribution and homogeneity of variances, one-way analysis of variance (ANOVA) followed by the least significant differences (LSD) test was used to analyze multiple groups, and independent samples Student’s t-test was used to determine statistical significance between two groups; data are expressed as the mean ± standard deviation ($\overline{x}$  ± SD). For non-normally distributed data, the non-parametric Kruskal–Wallis test was used to compare differences among groups; data are expressed as median (interquartile range, IQR) [M (P25, P75)]. Statistical significance was set at *P* < 0.05.

## Results

### Mice at 10 months of age showed ovarian dysfunction associated with aging

**Fig. 1:**
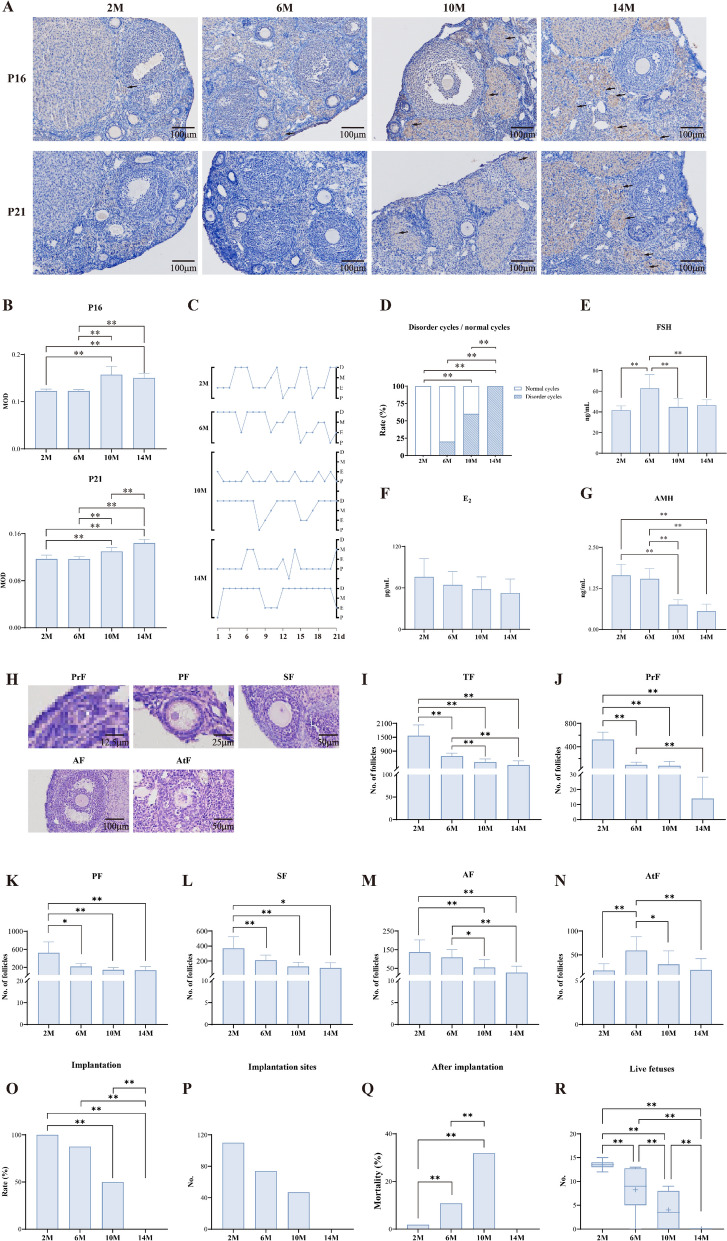
Mice at 10 months of age showed ovarian dysfunction associated with aging. **A** IHC staining of P16 and P21 in ovarian tissue obtained from female mice at 2, 6, 10, and 14 months old representatively (*n* = 6 mice/group). Black arrows indicate P16 and P21-positive cells. Scale bar = 100μm. **B** Mean optical density (MOD) results of IHC staining for P16 and P21. **C** Typical estrous cycle line graphs of mice in each group. **D** Estrous cycle disorder rate (*n* = 10 mice/group). **E**–**G** The serum levels of FSH, E_2_ and AMH assessed by ELISA (*n* = 6 mice/group). **H** Representative images of follicle classification by HE staining. PrF: primordial follicles; PF: primary follicles; SF: secondary follicles; AF: antral follicles; AtF: atretic follicles. **I**–**N** Quantitative analysis of follicle numbers (*n* = 10 mice/group). **O**-**R**. Reproductive performance in paired mating assays (*n* = 8 mice/group). ^**^P < 0.01, ^*^P < 0.05

To assess age-related changes in the ovaries, we collected ovarian tissues from female mice aged 2 (2 M), 6 (6 M), 10 (10 M), and 14 months (14 M). The expression of the cellular senescence markers P16 and P21 was indicative of a gradual increase in the senescent signature of the ovaries (Fig. 1A, B). Regarding the estrous cycle, which is a direct indicator of ovarian function, 2 M mice exhibited a regular 4–5-day cycle with four distinct periods: P, E, M, and D. The 6 M, 10 M, and 14 M groups showed varying degrees of cycle lengthening. Notably, 60% of the 10 M mice developed cyclical irregularities with prolonged D or P/E periods, whereas the 14 M mice experienced a complete cycle disorder characterized by persistent D or E periods (Fig. 1C, D). Serum hormone analysis revealed age-dependent effects; the 6 M mice showed significantly elevated FSH levels compared with the 2 M group, whereas both the 10 M and 14 M groups demonstrated decreased FSH levels. Serum E_2_ levels markedly declined with age in all groups. Additionally, the 10 M and 14 M mice exhibited reduced AMH concentrations compared with the 2 M and 6 M groups (Fig. 1E–G). The evaluation of follicles at various developmental stages revealed that, as age increased, the quantity of total, primordial, primary, secondary, and antral follicles in the ovaries of mice declined, particularly in the 10 M and 14 M groups (Fig. 1H–M). Additionally, compared with the 6 M group, the numbers of atretic follicles in the 10 M and 14 M groups were significantly lower (Fig. 1N). Fertility test outcomes further demonstrated an age-related decline, as both the average implantation rate and total number of implantation sites decreased significantly with advancing age, with no embryos implanted in the 14 M group. Concurrently, after-implantation mortality increased markedly in the 6 M and 10 M groups compared with the 2 M group, corresponding to reduced numbers of viable E18.5 fetuses (Fig. 1O–R). Collectively, these findings indicated that, beyond 10 months of age, female mice exhibited disrupted estrous cyclicity, aberrant serum hormone profiles, and ovarian microstructural alterations; all of which were indicative of substantial reproductive decline. This phenotype parallels reproductive aging observed in 35-year-old women. Investigating the biological events during this critical transition period might unveil the molecular mechanisms underlying reproductive senescence.

### Moxibustion improved ovarian function and fertility outcomes in 10-month-old mice

**Fig. 2:**
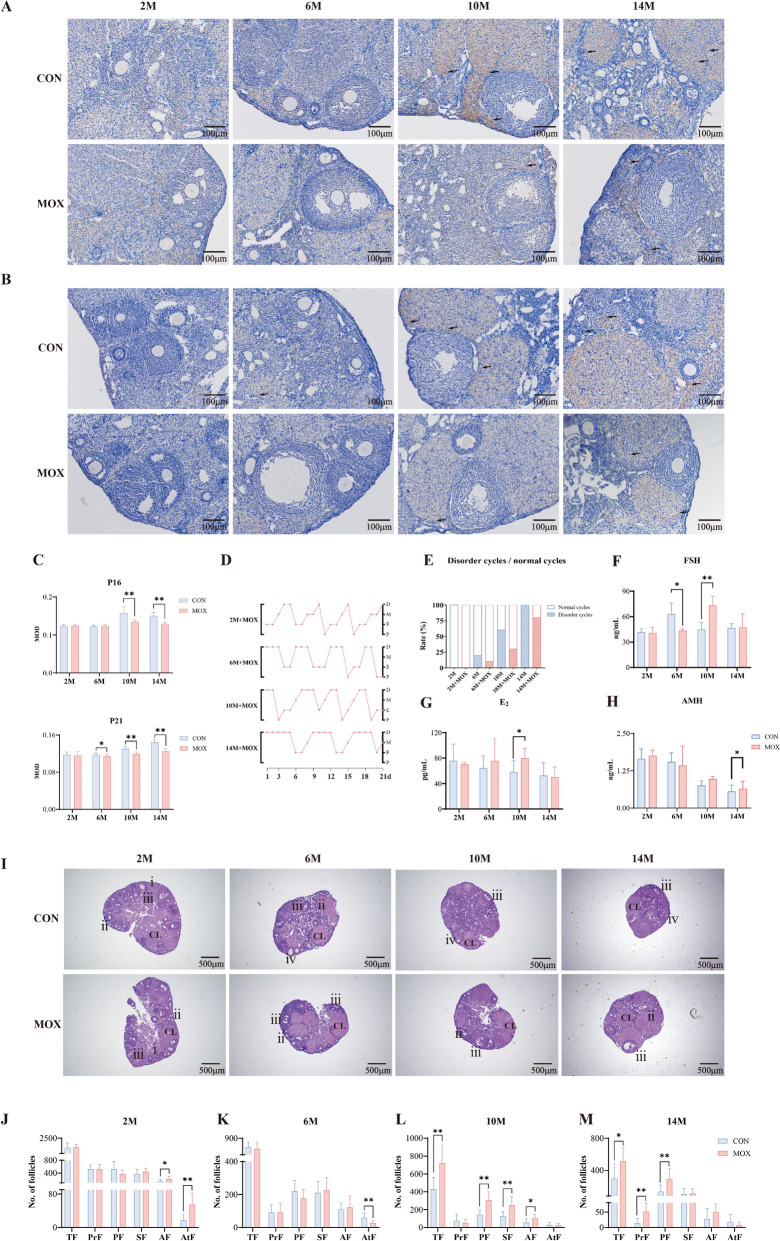
Moxibustion improved ovarian function in 10-month-old mice. **A**, **B** IHC staining of P16 and P21 in ovarian tissue obtained from CON and MOX groups at different months (*n* = 6 mice/group). Black arrows indicate P16 and P21-positive cells. Scale bar = 100μm. **C** MOD results of IHC staining for P16 and P21. **D** Typical estrous cycle line graphs of mice in MOX groups. **E** Estrous cycle disorder rate (*n* = 10 mice/group). **F**–**H** The serum levels of FSH, E_2_, and AMH assessed by ELISA (*n* = 6 mice/group). **I** Ovarian morphology by HE staining. i: primary follicles; ii: secondary follicles; iii: antral follicles; iv: atretic follicles; CL: corpus luteum. Scale bar = 500μm. **J**–**M** Quantitative analysis of follicle numbers (*n* = 10 mice/group). ^**^P < 0.01, ^*^P < 0.05

To ascertain the most effective intervention period for ovarian function improvement, the 21-day moxibustion treatments were administered at the aforementioned experimental time points (2, 6, 10, and 14 months of age). Following moxibustion intervention, we observed a statistically significant reduction in the MOD of P16-positive cells at 10 and 14 months of age (10 M + moxibustion [MOX] vs. 10 M, 14 M + MOX vs. 14 M) and P21-positive cells at 6, 10, and 14 months of age (6 M + MOX vs. 6 M, 10 M + MOX vs. 10 M, 14 M + MOX vs. 14 M) (Fig. 2A–C). Compared with mice in the control (CON) group, the incidence of disordered estrous cycles was reduced from 20% to 10% in 6-month-old mice (6 M + MOX vs. 6 M), 60% to 30% in 10-month-old mice (10 M + MOX vs. 10 M), and 100% to 80% in 14-month-old mice (14 M + MOX vs. 14 M) (Fig. 2D, E). Serum FSH levels decreased in the 6 M + MOX group, whereas its levels were elevated, with increases in E_2_ and AMH concentrations, in the 10 M + MOX group (Fig. 2F–H). The number of total and primary follicles increased in the 10 M + MOX and 14 M + MOX groups. Specifically, the number of primordial follicles increased in the 14 M + MOX group, and the number of secondary follicles increased in the 10 M + MOX group (Fig. 2I–M).

**Fig. 3:**
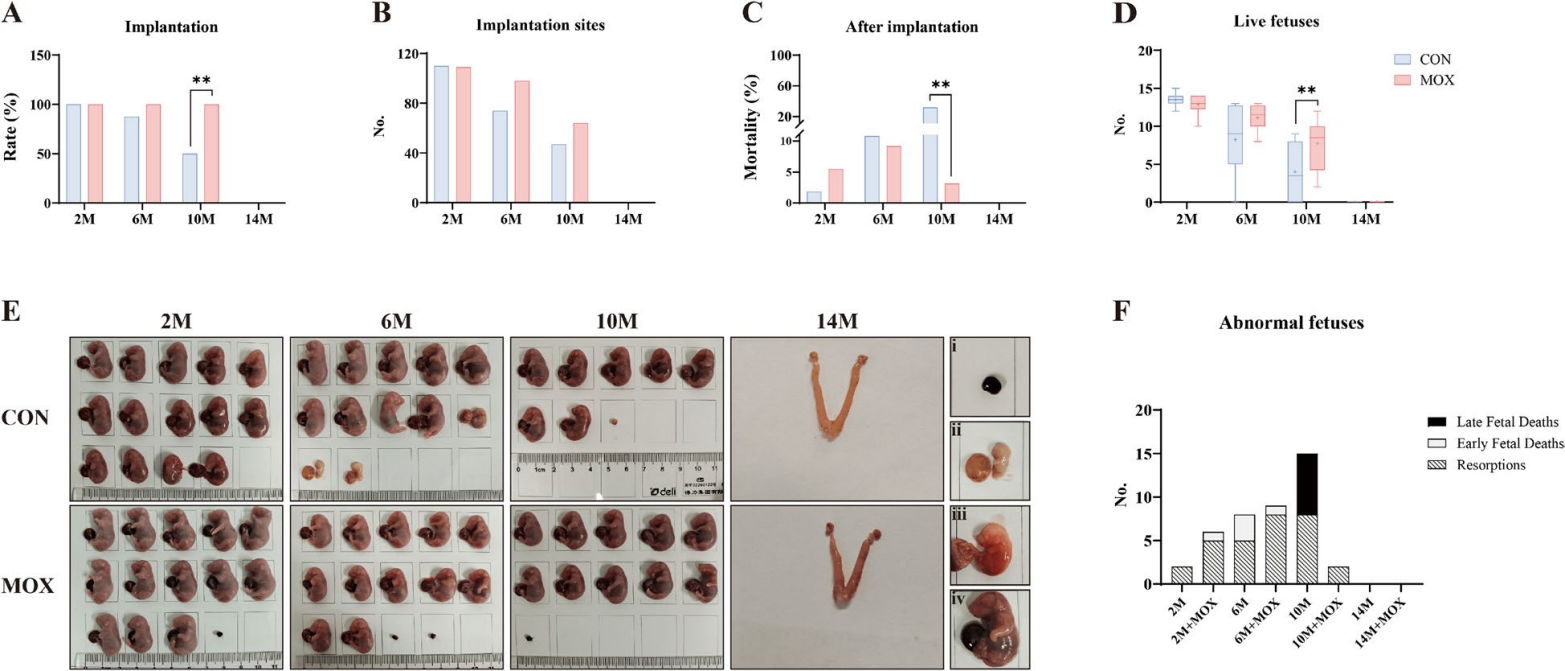
Moxibustion ameliorated fertility outcome in 10-month-old female mice. **A** The number of implantation. **B** The rate of implantation sites. **C** The mortality rate increase after implantation. **D** The number of live fetuses (–: median, top of box: P75, bottom of box: P25, +:  mean value). **E** The fetuses at E18.5. i: resorptions, ii: early fetal deaths, iii: late fetal deaths, iv: live fetuses. **F** The number of abnormal fetuses. Compared with the CON groups, ^**^P < 0.01; compared among the MOX groups, ^##^*P* <0.01; compared with the control group of the same age,^△△^*P*  <  0.01

According to the results of fertility experiments, moxibustion enhanced the rate of implantation and number of live fetuses and reduced implantation mortality in the 10 M + MOX group (Fig. 3A–D). Notably, moxibustion decreased embryonic abnormalities in 10-month-old mice, characterized by the complete absence of late fetal death and a significantly reduced number of resorption sites in the 10 M + MOX group (Fig. 3E, F). The results indicated that moxibustion was most beneficial at 10 months of age, mitigating age-related declines in ovarian reserve and fertility associated with aging.

### Moxibustion improved age-related oocyte quality decline

**Fig. 4:**
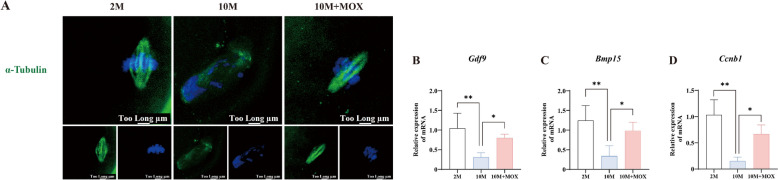
Moxibustion improved age-related oocyte quality decline **A** Spindle assembly in oocyte. **B**–**D** The relative expression of *Gdf9*, *Bmp15*, and *Ccnb1* mRNA in oocytes. ^**^P < 0.01, ^*^P < 0.05

Oocyte quality is a key limiting factor in female fertility. Given the significant effects of moxibustion on 10-month-old mice, we then assessed oocyte quality among 2M, 10M, and 10M + MOX groups. The analysis focused on meiotic spindle integrity, chromosomal alignment, and the expression levels of oocyte-specific factors (growth differentiation factor 9, *Gdf9*; bone morphogenetic protein 15, *Bmp15*) and cyclin B1(*Ccnb1*), a key regulator of spindle formation[19]. Immunofluorescence quantification revealed that oocytes in the 2 M group exhibited symmetrical fusiform spindles with acentrosomal poles and orderly metaphase plate alignment (Fig. 4A). In contrast, 10 M oocytes showed spindle disassembly, irregular morphology, and disorganized hyper-condensed chromosomes displaced from the equatorial plate, indicating age-related damage. Consistent with these defects, the expression levels of *Gdf9*, *Bmp15*, and *Ccnb1* were significantly downregulated in 10 M oocytes (Fig. 4B–D). Moxibustion intervention restored spindle assembly and upregulated the mRNA expression of *Gdf9*, *Bmp15*, and *Ccnb1*, demonstrating its efficacy in ameliorating age-associated declines in oocyte quality.

### Moxibustion promoted mitochondrial quality in oocytes of 10-month-old mice

**Fig. 5:**
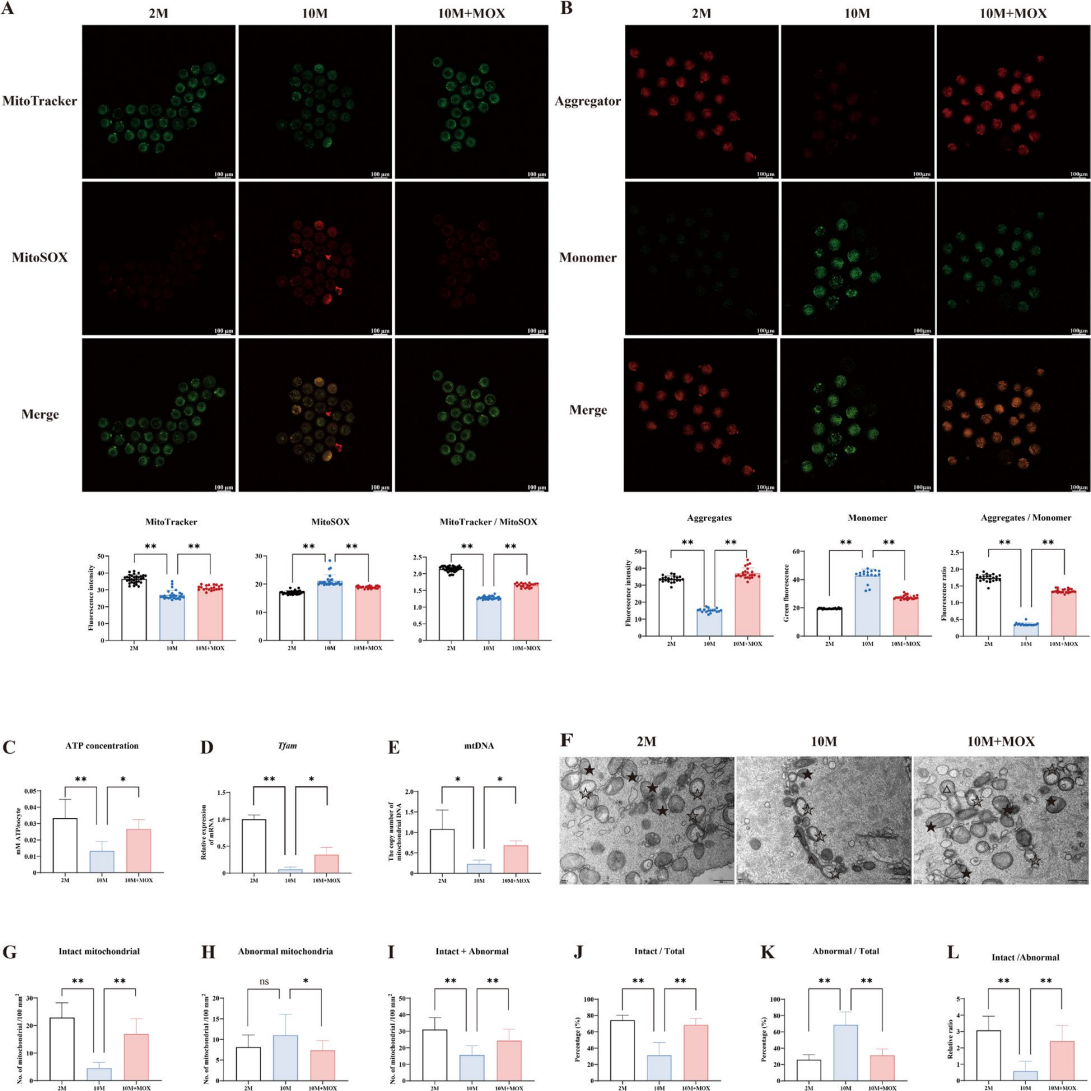
Moxibustion promoted mitochondrial quality in oocytes of 10-mouth-old mice. **A** Distribution of mitochondria and release of ROS (n = 3–6 mice/group, each dot represents an individual oocyte). **B** Detection of MMP. **C** ATP concentration. **D** *Tfam* mRNA expression. **E** The copy number of mtDNA. **F** The morphology of mitochondria by transmission electron microscopy. ★: intact mitochondria, ☆: vacuole mitochondria, △: dumbbell-shaped mitochondria, scale bar = 500nm. **G**–**I** The number of intact, abnormal and total mitochondria. **J**, **K** The percentage of intact, abnormal mitochondria. **L** The ratio of intact and abnormal mitochondrial. **P < 0.01, *P < 0.05

Mitochondrial dysfunction is a crucial characteristic of ovarian aging [20]. MitoTracker was used to label mitochondria; the results demonstrated a significant reduction in mitochondrial fluorescence intensity within oocytes of the 10 M group compared with the 2 M control group. Notably, moxibustion increased mitochondrial fluorescence intensity and facilitated a more uniform distribution of mitochondria. To explore ROS production in the mitochondria, we co-stained the mitochondria with a mitochondrial superoxide indicator. The results indicated that moxibustion reduced the ROS levels in the mitochondria, thereby increasing their ratio (Fig. 5A). Moxibustion therapy also enhanced MMP and ATP levels (Fig. 5B, C). Moxibustion increased mtDNA copy number and mitochondrial transcription factor A (*Tfam*) mRNA expression (Fig. 5D, E), which are representative indicators of mitochondrial quantity. In line with this, we observed that the total number of mitochondria significantly increased in the 10 M + MOX group; specifically, the number and rate of intact mitochondria increased (Fig. 5F–L).

### Moxibustion activated mitochondrial biogenesis and enhanced mitochondrial quantity via PGC-1α

**Fig. 6:**
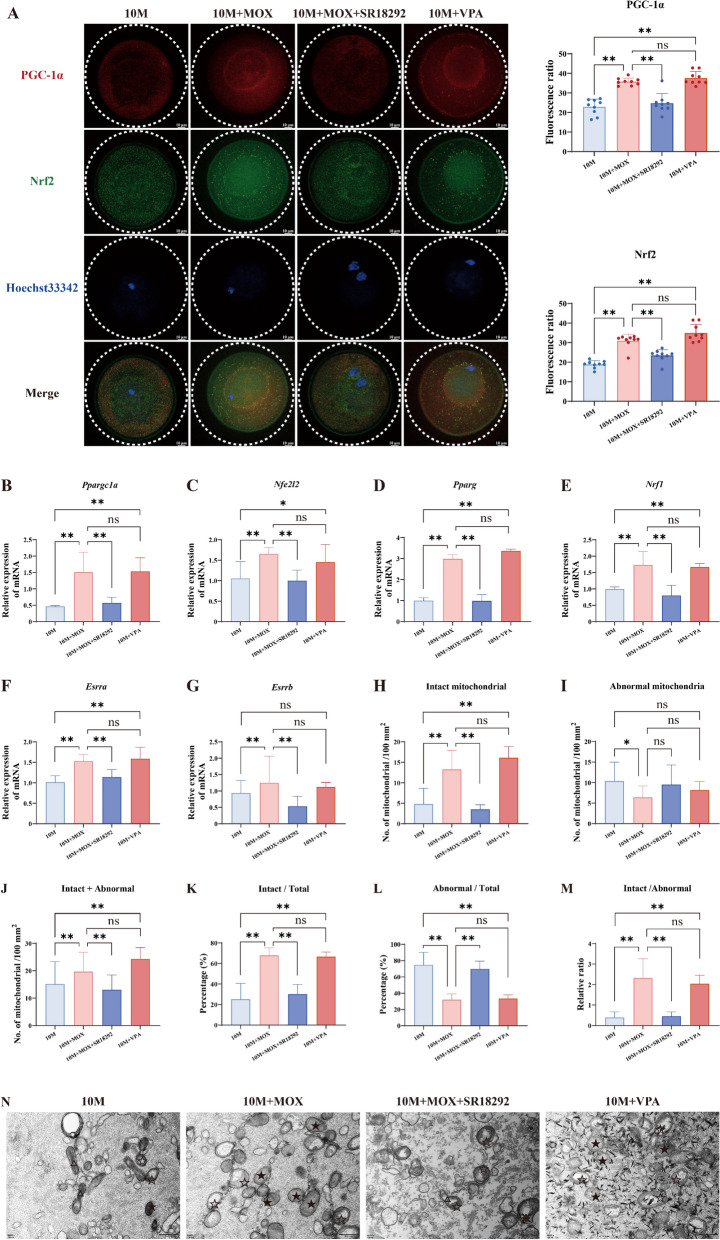
Moxibustion activated mitochondrial biogenesis and enhanced mitochondrial quantity via PGC-1α. **A** Immunofluorescence staining and the relative fluorescence intensities of PGC-1α and Nrf2 in oocytes. **B**–**G** The relative expression of *Ppargc1a* (PGC-1α), *Nfe2l2* (Nrf2), *Pparg* (PPARγ), *Nrf1*, *Esrra* (ERRα), and *Esrrb* (ERRβ) mRNA in oocytes. **H**–**J** The number of intact, abnormal, and total mitochondria. **K**, **L** The percentage of intact and abnormal mitochondria. **M** The ratio of intact and abnormal mitochondria. **N** The morphology of mitochondria by transmission electron microscopy. ★: intact mitochondria, ☆: vacuole mitochondria, △: dumbbell-shaped mitochondria, scale bar = 500nm. **P < 0.01, *P < 0.05

PGC-1α served as a pivotal regulator in the process of mitochondrial biogenesis [21], possessing the capability to activate crucial mitochondrial transcription factors, including peroxisome proliferator-activated receptors (Pparg), estrogen-related receptors (Esrra, Esrrb), and Nuclear respiratory factor 1 (Nrf1). It then operates in concert with nuclear factor erythroid 2-related factor 2 (Nrf2) and exerts an indirect influence on mtDNA replication [22]. In the 10 M group, the PGC-1α protein was localized at the cellular edge, whereas Nrf2 was dispersed throughout the cytoplasm, both exhibiting relatively low fluorescence intensities. After administering the PGC-1α agonist valproic acid sodium (VPA), PGC-1α demonstrated a tendency to aggregate near the nucleus and even showed a propensity to enter the nucleus, accompanied by a significant increase in fluorescence intensity. Similarly, Nrf2 displayed intense perinuclear fluorescence localization, with a notable elevation in fluorescence intensity (Fig. 6A). Notably, oocytes in the 10 M + MOX group exhibited comparable expression patterns of PGC-1α and Nrf2 to those in the 10 M + VPA group. However, the transcription levels of other cytosolic factors related to mitochondrial biogenesis were significantly upregulated (Fig. 6B–G). These findings were corroborated by mitochondrial counting under a transmission electron microscopy (Fig. 6H–N), which confirmed that moxibustion promoted mitochondrial biogenesis.

Subsequently, we employed SR18292, a PGC-1α antagonist, to explore the impact of moxibustion administration on mitochondrial biogenesis in the oocyte. The oocytes in the 10 M + MOX + SR18292 group exhibited localization patterns of PGC-1α and Nrf2 proteins that were similar to that in the 10 M group. The fluorescence intensity of these two proteins was notably reduced compared with the 10 M + MOX group (Fig. 6A). Both the number and percentage of intact mitochondria decreased in the 10 M + MOX + SR18292 group compared with those in the 10 M + MOX group (Fig. 6H, K). This further substantiated the proposition that moxibustion might activate mitochondrial biogenesis through the PGC-1α/Nrf2 pathway, thereby enhancing the mitochondrial quantity in aging oocytes.

### Mitochondrial biogenesis activated by moxibustion partially improved mitochondrial function and rescued maternal age-related oocyte quality decline

**Fig. 7:**
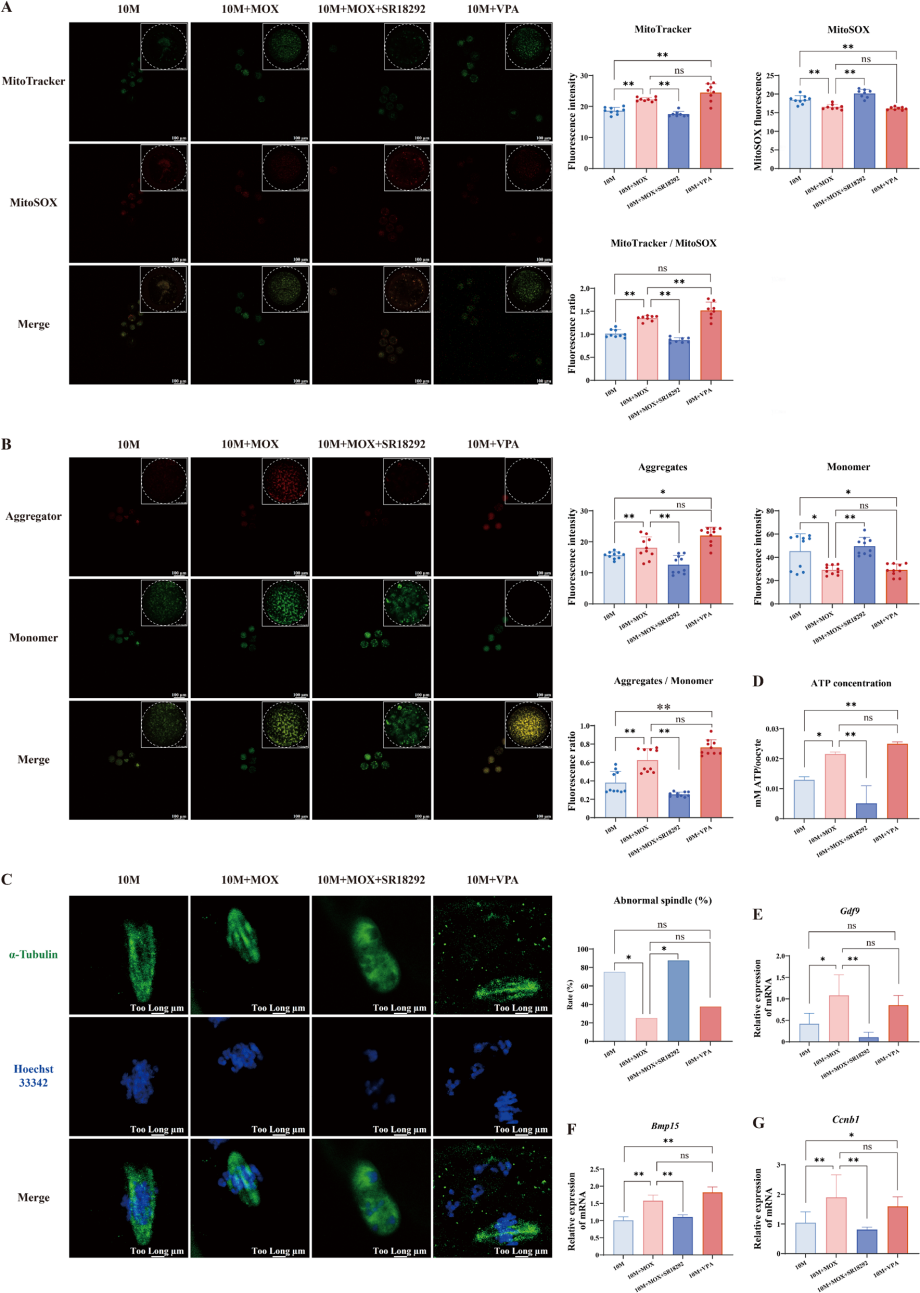
Mitochondrial biogenesis activated by moxibustion partially improved mitochondrial function and rescued maternal age-related oocyte quality decline. **A** Distribution of mitochondria and release of ROS. **B** Detection of MMP. **C** Spindle assembly in oocytes. **D** ATP concentration. **E**–**G** The relative expression of *Gdf9*, *Bmp15*, and *Ccnb1* mRNA in oocytes. ^**^P < 0.01, ^*^P < 0.05

To further ascertain the impact of moxibustion-induced mitochondrial biogenesis on oocytes, we examined changes in mitochondrial function and oocyte quality. Through the activation of mitochondrial biogenesis, moxibustion suppressed ROS production while augmenting MMP and ATP content (Fig. 7A, B, D). Consequently, moxibustion facilitated spindle assembly, ensured accurate chromosome segregation, and elevated the secretion of *Gdf9*, *Bmp15*, and *Ccnb1* (Fig. 7C, E–G).

## Discussion

Age is an independent risk factor affecting female fertility and pregnancy outcomes. The success rate of ART decreases with increasing age; the age-related decline in pregnancy rates remains a significant challenge for ART [1, 23]. Hence, concentrating on mitigating female reproductive senescence is imperative, thereby preserving fertility and hormonal functions associated with aging. Moxibustion possesses a long history of clinical practice in the fields of health preservation and aging delaying, and it serves as a vital therapeutic approach for improving ovarian function and enhancing female fertility [15–17, 24]. Therefore, this study focused on the entire reproductive cycle of female mice, and the optimal timing for moxibustion intervention to achieve the most significant and efficient improvement in mitigating ovarian aging was identified as 10 months of age. Furthermore, it centered on oocytes to explore the mitochondrial mechanisms through which moxibustion administration improved oocyte quality.

Moxibustion has unique advantages in slowing down the aging process and maintaining the physiological functions of the ovaries. In female rats, moxibustion improved naturally aging ovarian function by inhibiting apoptosis and upregulating antioxidant defenses in the ovary [15]. In addition, moxibustion had estrogen-like effects and regulated circulating E_2_ and its receptor levels and inhibited ovarian apoptosis in senescent female rats [25]. In perimenopausal women, moxibustion also improves quality of life, regulates the levels of sex hormones and AMH, improves ovarian reserve function, and delays ovarian aging [26].

Female fertility in humans begins to decline at the age of 32 and accelerates after the age of 37 [27]. In mice, ovarian reserve gradually decreases starting from 6 to 8 months old, with the number of primordial follicles at 10 months being only half of that at 4 months, and the number of growing follicles decreases to two-thirds [28, 29]. In this study, mice aged 2 to 6 months are considered equivalent to 20–30-year-old women, mice aged 10 and 14 months are considered middle-aged and equivalent to 33–38-year-old and 46–47-year-old women, respectively [30, 31]. Ovarian follicles of all classes, including primordial, primary, secondary, and antral follicles, progressively decreased in number in 6 M, 10 M, and 14 M mice, accompanied by a continuous increase in the rate of disordered estrous cycles. Moxibustion had the most beneficial effects on fertility when given to 10 M mice, which was specifically manifested as an improvement in the live fetus number by reducing embryonic mortality. However, this beneficial effect on fertility was not observed in 14 M mice. It indicated that moxibustion had certain limitations in its effect on ovarian function and emphasized that intervention at the appropriate age was crucial for maximizing the improvement of aging ovarian function. This provided experimental reference for the selection of the intervention timing of moxibustion in the clinic.

Therapeutic interventions in the clinic have been developed to support the growth of rare follicles within the ovary and extend reproductive capacity. However, little attention has been paid to the important endocrine role of ovarian hormones in maintaining overall health during the aging process. The declines in estrogen levels affect multiple systemic functions [32], not only causing autonomic nervous system dysfunction (such as hot flashes, night sweats, etc.), urogenital symptoms, and cognitive impairments [33–36], which adversely impact quality of life, but also increasing the risk of cardiovascular disease and osteoporosis [37–39], among others. Nevertheless, moxibustion could not only promote follicle growth but also maintain ovarian hormone secretion function as much as possible. In other words, moxibustion supported follicle growth by improving ovarian endocrine function. Our results indicated that moxibustion significantly increased the serum E_2_ level in 10 M mice and improved ovarian poor response with a concomitant increase in FSH concentration. This was consistent with clinical reports that moxibustion administration improved estrogen levels in patients with ovarian function decline [40] and alleviated perimenopausal symptoms such as hot flashes and insomnia in women [41].

Targeting oocyte quality may be a promising new therapeutic direction to improve female fertility. Therefore, the present study observed the quality of oocytes. The results suggested that, in parallel to supporting the growth of rare follicles, moxibustion administration was also aimed at addressing the critical issue of poor oocyte quality. 10 M mice exhibited defects in oocyte quality with age, typically manifested by increased spindle abnormalities and chromosome misalignment. Moxibustion intervention significantly improved this situation. Additionally, the improved oocyte quality was consistent with the increased number of implantation sites and live fetuses.

Mitochondria are emerging as major intracellular targets for intervention because of their critical role in developmental competence following fertilization, and deficiencies in oocyte mitochondrial function were a hallmark of female reproductive aging [20, 42]. In present study, the results showed that the mitochondrial protective mechanism was inadequate in preserving the homeostatic balance of oocytes because of the persistent stress associated with aging. Specifically, oocytes in the 10 M group exhibited a heterogeneous distribution of mitochondrial function, characterized by a decline in MMP and reduced ATP production. Meanwhile, the results of mitochondrial counting showed a significant decrease in the total number of mitochondria and the number of intact mitochondria, suggesting that mitochondrial biogenesis is disrupted during the maturation stage of oocytes in the 10 M group. Mitochondrial biogenesis, as a crucial aspect of mitochondrial quality control, enhances mitochondrial function through the generation of new mitochondria. Mitochondrial biogenesis is indispensable for sustaining oocyte function. Throughout the process of oocyte cellular maturation, from the germinal vesicle (GV) stage to the mature MII stage, there is a significant increase in the number of mitochondria, ultimately culminating in hundreds of thousands of mitochondria within a single oocyte prior to fertilization. This quantity remains constant during the period of fertilization until embryo implantation, whereupon mitochondrial biogenesis resumes. These mitochondria are pivotal in generating sufficient ATP to facilitate successful fertilization and nurture the development of the preimplantation embryo sac [43]. Inadequate mitochondrial biogenesis during oocyte maturation has been linked to fertilization failure and abnormal early embryo development [4, 44]. Therefore, impaired mitochondrial biogenesis may significantly contribute to the decline in mitochondrial function and oocyte quality in the 10 M group. Notably, moxibustion administration increased the number of functional mitochondria and improved the quality of oocytes in the 10 M + MOX group. Further determining whether moxibustion could improve oocyte quality by activating mitochondrial biogenesis is necessary.

PGC-1α emerges as a crucial factor in mitochondrial biogenesis, orchestrating transcription to augment mitochondrial mass and facilitate cell adaptation to heightened energy requirements [45]. We conducted relevant rescue experiments focusing on PGC-1α. After upregulating PGC-1α to induce mitochondrial biogenesis, mitochondrial function––including mitochondrial distribution, ROS levels, MMP, and ATP content––was significantly enhanced, further improving oocyte spindle assembly and quality. Importantly, moxibustion exhibited similar efficacy to PGC-1α agonists in promoting mitochondrial biogenesis and, subsequently, enhancing mitochondrial function and improving oocyte quality. Furthermore, the positive effects of moxibustion were inhibited when a PGC-1α antagonist was used. The above results supported the hypothesis that moxibustion might improve the quality of aging oocytes by activating mitochondrial biogenesis.

While the present study demonstrates that moxibustion can delay female reproductive aging, a lack of a comprehensive evaluation of the long-term effects of moxibustion on ovarian function in reproductively aging mice remains lacking, and whether this intervention plays a role in male reproductive aging has yet to be explored. Future research could address these gaps by increasing the number of mating cycles and litters, as well as by including male studies to assess whether moxibustion exerts similar benefits on reproductive aging in both sexes. Additionally, mating experiments or in vitro fertilization studies should be conducted to assess early embryonic development competence after confirming that moxibustion enhances oocyte quality via mitochondrial biogenesis. It is also important to note that the present findings are based on an animal model, and further validation in human trials is needed to determine the translational relevance of moxibustion for reproductive aging in clinical settings.

## Conclusion

This study demonstrated that moxibustion maximized the enhancement of ovarian function in 10-month-old female mice, an effect primarily attributed to the improvement in oocyte quality. The underlying issue may involve diminished mitochondrial biogenesis in oocytes with aging. Moxibustion might extend fertility in reproductively aging mice potentially through enhanced mitochondrial biogenesis. Together, moxibustion may have valuable applications for extending women's reproductive lifespan and reproductive choices.

## Supplementary Information

Additional file 1.

Additional file 2.

## Data Availability

No datasets were generated or analysed during the current study.

## Abbreviations

ART: Assisted reproductive technology
AMH: Anti-Mullerian hormone
Bmp15: Bone morphogenetic protein 15
Ccnb1: Cyclin B1
E_2_: Estradiol
Esrra: Estrogen-related receptor α
Esrrb: Estrogen-related receptor β
FSH: Follicle stimulating hormone
Gdf9: Growth differentiation factor 9
IHC: Immunohistochemical
MII: Metaphase II
MMP: Mitochondrial membrane potential
MOX: Moxibustion
mtDNA: Mitochondrial DNA
Nrf1: Nuclear respiratory factor 1
Nrf2/Nfe2l2: Nuclear factor erythroid 2-related factor 2
PGC-1α/Ppargc1a: Peroxisome proliferator-activated receptor γ coactivator 1α
Pparg: Peroxisome proliferator-activated receptor α
ROS: Reactive oxygen species
Tfam: Mitochondrial transcription factor A
VPA: Valproic acid sodium
