# Supplementary material for: Moxibustion delays ovarian aging by regulating mitochondrial biogenesis and improving oocyte quality
Source: Chin Med. 2026 Apr 10;21:115. doi: 10.1186/s13020-026-01375-3 (PMC13067729; doi:10.1186/s13020-026-01375-3)
Supplement: Supplementary file 1 — Additional file 1. [file 13020_2026_1375_MOESM1_ESM.doc]

**Experimental groups**

Experiment 1: A total of 144 2-month-old female mice with normal estrous cycles were divided into two groups: the control (CON) and moxibustion (MOX) groups. At each time point (2, 6, 10, and 14 months of age), 18 mice per group were randomly selected; 10 mice were humanely euthanized under terminal anesthesia for sample collection, while the remaining 8 mice underwent fertility testing. The MOX group received 21-day moxibustion treatment before sample collection and fertility assessments at the same point.

Experiment 2: Based on the aforementioned experimental process, female mice aged 10 months were employed as a model of natural reproductive aging. A total of 44 mice of 10-month-old were randomly assigned to two groups: a model (10M) group and a moxibustion (10M+MOX) group, with 22 mice in each group. Additionally, 22 mice aged 2 months were designated as the control (2M) group. Following moxibustion intervention, oocytes at the MⅡ stage were harvested from each group.

Experiment 3: The inhibition of peroxisome proliferator‐activated receptor γ coactivator 1α (PGC-1α) SR18292 (S8528, Selleck) and the agonist of PGC-1α Valproic Acid sodium (VPA, S1168, Selleck) were used in rescued experiments. 10-month-old female mice were randomly divided into four groups: 10M (*n*=40), 10M+MOX (*n*=40), 10M+MOX+SR18292 (*n*=40), 10M+VPA (*n*=40). The mice in the 10M+MOX+SR18292 group were administered intraperitoneal injections of SR18292 (45 mg/kg dissolved in DMSO) twice a week for 21d. The mice in the 10M+VPA group were administered intraperitoneal injections of VPA (200 mg/kg dissolved in saline) daily for 21d. Equivalent frequencies and volumes of DMSO were used as the controls. Furthermore, the mice in the 10M+MOX and 10M+MOX+SR18292 groups received 10-min moxibustion 1h after injections.


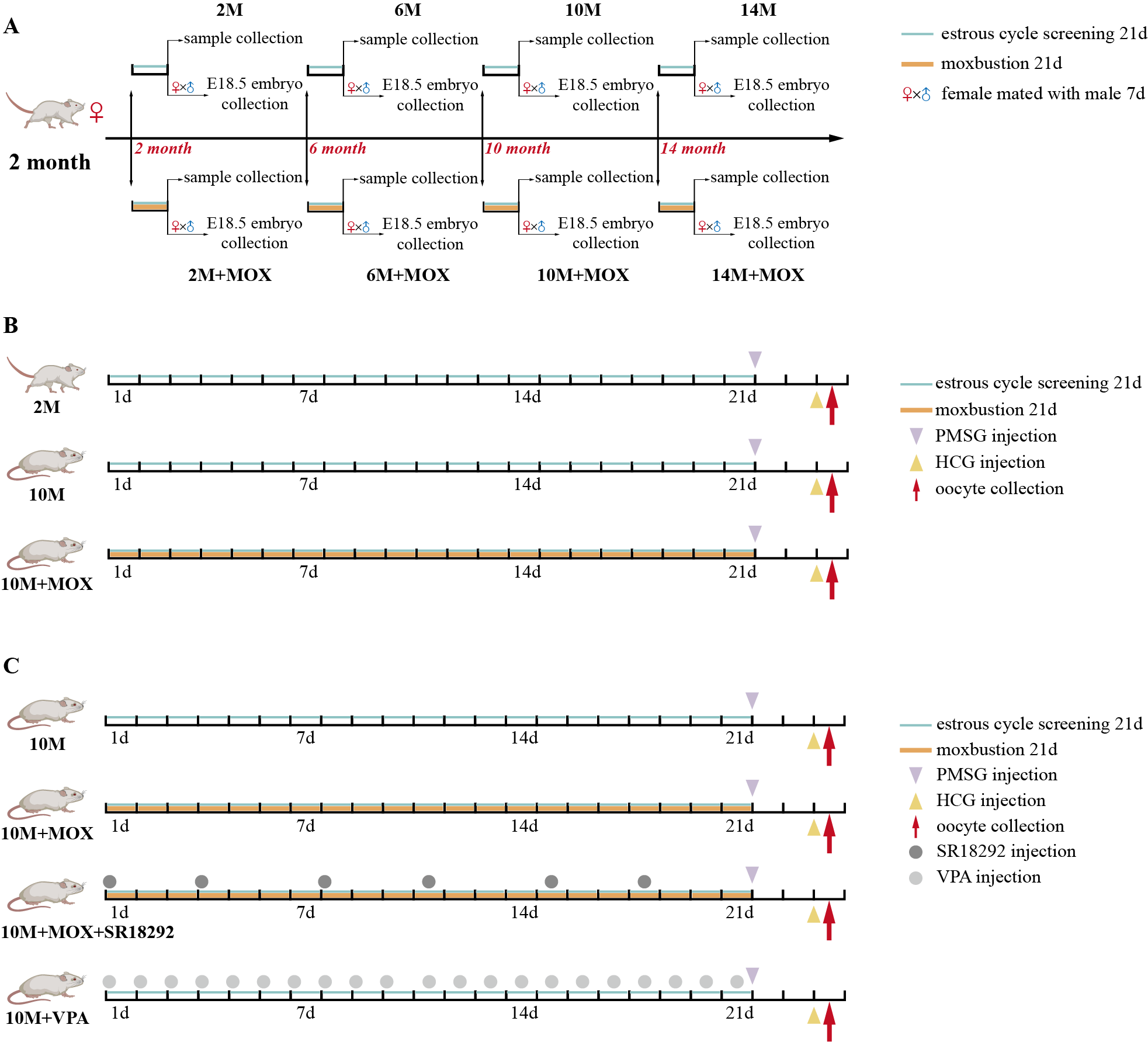


**Fig S1** Experimental groups and interventions. **(A)** Experimental 1. **(B)** Experimental 2. **(C)** Experimental 3.
