# Supplementary material for: Moxibustion delays ovarian aging by regulating mitochondrial biogenesis and improving oocyte quality
Source: Chin Med. 2026 Apr 10;21:115. doi: 10.1186/s13020-026-01375-3 (PMC13067729; doi:10.1186/s13020-026-01375-3)
Supplement: Supplementary file 2 — Additional file 2. [file 13020_2026_1375_MOESM2_ESM.doc]

**Table S1. The list of primers used for quantitative RT-PCR.**

| **Gene** | **Sequence(5'-3')** | | **Length(bp)** |
| --- | --- | --- | --- |
| *Gapdh* | Forward | GGTTGTCTCCTGCGACTTCA | 183 |
|  | Reverse | TGGTCCAGGGTTTCTTACTCC |  |
| *Gdf9* | Forward | CTCTGCCTCTTCCTCCTCCACTG | 103 |
|  | Reverse | GGTGAATGAGTACGGTGCTCTTGG |  |
| *Bmp15* | Forward | GAATCGCAAGGGACGGAGTGTTC | 120 |
|  | Reverse | AGTAGCAAGAAGGCAACATCCAAGG |  |
| *Ccnb1* (cyclin B1) | Forward | CTCACCGACGAGGAACTGAATGC | 84 |
|  | Reverse | CTCACCGACGAGGAACTGAATGC |  |
| mtDNA | Forward | TATGATTACTTCTGCCAGCCTGAC | 114 |
|  | Reverse | CTGATACTAATTCTGATTCTCCTTCTGTC |  |
| *Tfam* | Forward | GGGGTCTTGTCTGTATTCCGAAGTG | 99 |
|  | Reverse | TGGGTAGCTGTTCTGTGGAAAATCG |  |
| *Ppargc1a* (PGC-1α) | Forward | GTGCCACCGCCAACCAAGAG | 142 |
|  | Reverse | TTCCTCGTGTCCTCGGCTGAG |  |
| *Nfe2l2*  (Nrf2) | Forward | TGCCACCGCCAGGACTACAG | 109 |
|  | Reverse | GCGTGCTCAGAAACCTCCTTCC |  |
| *Pparg*  (PPARγ) | Forward | CTTCACGATGCTGTCCTCCTTGATG | 112 |
|  | Reverse | GATGTCACAGAACGGCTTCCTCAG |  |
| *Esrra*  (ERRα) | Forward | GGCGGACGGCAGAAGTACAAAC | 133 |
|  | Reverse | GATGCGACACCAGAGCGTTCAC |  |
| *Esrrb*  (ERRβ) | Forward | CATTGATGCCCTCAGCCACCAC | 21 |
|  | Reverse | ACATAGGCGGCGAGTCCAGAC |  |
| *Nrf1* | Forward | TGAATTACTCTGCTGTGGCTGATGG | 96 |
|  | Reverse | CCTCTGATGCTTGCGTCGTCTG |  |
